# Influence of concomitant percutaneous transluminal angioplasty with percutaneous coronary intervention on clinical outcomes of stable lower extremity artery diseases

**DOI:** 10.1038/s41598-022-16631-3

**Published:** 2022-07-29

**Authors:** Yonggu Lee, Byung-Sik Kim, Jeong-Hun Shin, Woohyeun Kim, Hyungdon Kook, Hwan-Cheol Park, Minae Park, Sojeong Park, Young-Hyo Lim

**Affiliations:** 1 Division of Cardiology, Department of Internal Medicine, Hanyang University College of Medicine, 222 Wangsimni-ro, Sungdong-gu, Seoul, 04763 Republic of Korea; 2 Department of Cardiology, Hanyang University Guri Hospital, Gyeong-Choon Street 153, Guri, Gyounggido Republic of Korea; 3 Data Science Team, Hanmi Pharmaceutical Company Limited, Seoul, South Korea

**Keywords:** Outcomes research, Cardiology, Medical research

## Abstract

Concomitant percutaneous transluminal angioplasty (PTA) at the time of percutaneous coronary intervention (PCI) is often performed because lower extremity artery disease (LEAD) commonly coincides with coronary artery disease. We investigated the impact of concomitant PTA on both cardiovascular and limb outcomes in the Korean National Health Insurance Service registry. Among 78,185 patients undergoing PCI, 6563 patients with stable LEAD without limb ischemia were included. After 1:5 propensity score matching was conducted, 279 patients in the PTA + PCI group and 1385 patients in the PCI group were compared. Multivariate Cox proportional hazard models showed that the risk of all-cause death was higher in the PTA + PCI group than in the PCI group, whereas the risks of myocardial infarction, repeat revascularization, stroke, cardiovascular death and bleeding events were not different between the 2 groups. In contrast, the risks of end-stage renal disease and unfavorable limb outcomes were higher in the PTA + PCI group. Mediation analyses revealed that amputation and PTA after discharge significantly mediated the association between concomitant PTA and all-cause death. Concomitant PTA was not associated with an increased risk of cardiovascular events but may increase the risk of all-cause death mediated by unfavorable renal and limb outcomes in patients with stable LEAD.

## Introduction

For decades, percutaneous transluminal angioplasty (PTA) has been widely selected as a treatment strategy for patients who have stable lower extremity artery disease (LEAD) without critical limb ischemia (CLI) to improve their lifestyle-limiting symptoms^1^. Patients with stable LEAD frequently present with multisite artery disease. Significant coronary artery disease (CAD) is found in up to 70% of patients presenting with LEAD, and LEAD is found in 16% of patients presenting with significant CAD^1^. Consequently, concomitant PTA at the time of percutaneous coronary intervention (PCI) is often performed for patients with significant CAD and symptomatic LEAD in current clinical practice^2,3^. These concomitant PTAs performed by skilled interventional cardiologists may be effective in rapidly improving lifestyle-limiting symptoms in patients with stable LEAD. However, concomitant endovascular procedures at the time of PCI would increase the exposure to contrast media and parenteral anticoagulants, which may increase the risk of renal impairment and bleeding^4^. Moreover, these procedures may promote systemic inflammatory responses through extensive injuries to the diseased vessels^5^, which may eventually result in coronary in-stent restenosis (ISR) during follow-up^6^. Because it is crucial to determine how concomitant PTA affects clinical outcomes following PCI, we investigated the impact of concomitant PTA on CV, limb and renal outcomes in patients who underwent PCI using the Korean National Health Insurance System (KNHIS) database.

## Methods

### Data source

This study is an observational retrospective study conducted using the Korean National Health Insurance Data Sharing Service (KNHISS) customized research database. The KNHISS is a KNHIS-operating data-sharing service that provides a sample cohort database as well as customized databases to facilitate the use of national health information for research purposes. Detailed information about the KNHISS customized database was described previously^7^ and is presented in Supplementary Data 1.

All procedures in this study protocol adhered to the ethical principles of the Declaration of Helsinki. The Institutional Review Board of Hanyang University Guri Hospital (GURI-2019-03-032) and the ethics committee of the KNIHS (NHIS-2019-1-561) reviewed and approved the protocol of this study. Informed consent from the subjects was exempted because the data were deidentified.

### Study population

From the KNHISS database, we included patients with stable LEAD who underwent PCI between January 2014 and December 2015. PTA was reimbursed within this period when a patient was (1) diagnosed with symptomatic LEAD and (2) presented with at least one lesion with stenosis  ≥ 70% in diameter. Patients who had undergone any prior PCI or PTA between January 2012 and December 2014 or those who had any diagnosis or reimbursement codes suggesting acute limb ischemia, CLI or limb amputations were excluded. Because of the research ethics policy declared by the KNHISS, only 30% of the entire population was randomly selected to produce the customized database for this study.

### Definitions of the clinical characteristics and outcomes

The estimated glomerular filtration rate (eGFR) was calculated using the Chronic Kidney Disease–Epidemiology Collaboration equation. Chronic kidney disease (CKD) was defined as eGFR ≤ 60 mL/min/1.73 m^2^. The follow-up duration was defined from the day of the first visit to the outpatient office after index PCI to the day when the first clinical event occurred, when the reimbursement claims stopped emerging in the database, or when 5 years had passed after index PCI. All patients were followed for at least 3 years. Comorbidities and clinical events were identified using KCD-7 codes. Charlson’s comorbidity index (CCI) was used to represent the severity of the comorbidities as previously described^8^. Detailed KCD-7 codes used to define the comorbidities and clinical outcomes are described in Supplementary Table 1. Myocardial infarction (MI) was defined as a new diagnosis of MI codes (I21-I24) during admission, stroke was defined as a new diagnosis of the stroke codes (I60-I64) during admission, and CV death was defined as death from CV disease (I20-I25, I42-I43, I50), cerebrovascular events (I60-I69) or peripheral vascular diseases (I70-I74).

Coronary revascularization was defined as a composite of PCI and coronary artery bypass surgery. Repeat coronary revascularization was defined as coronary revascularization after index hospitalization. A major adverse CV event (MACE) was defined as a composite of CV death, MI, stroke and repeat coronary revascularization. Bleeding events were defined as new diagnosis codes for bleeding events requiring admission or transfusion (Supplementary Table 1) based on the GUSTO (Global Utilization of Streptokinase and Tissue Plasminogen Activator for Occluded Coronary Artery) criteria^9^. End-stage renal disease (ESRD) was defined as a new diagnosis code for ESRD (N18.5). CLI was defined as a new diagnosis code for diabetic foot ulcer/gangrene or amputation of the lower extremities (Supplementary Table 1).

PCI and PTA were defined as insurance claim codes representing the procedures (Supplementary Table 2) with those representing the devices designated for their respective procedures, including plain angioplasty balloons, drug-coated balloons, bare-metal stents (BMSs) or drug-eluting stents (DESs) during the index hospitalization (Supplementary Table 3). In contrast, coronary artery bypass surgery was defined using the procedure codes only (Supplementary Table 2). Concomitant PTA at the time of PCI was defined as PTA performed during the same index hospitalization period when PCI was performed. PTA after discharge was defined as PTA during the first readmission after index hospitalization.

### Statistical analysis

Because the patients undergoing concomitant PTA at the time of PCI were expected to have more severe atherosclerotic CV diseases with more comorbidities than those undergoing PCI alone, we performed propensity score matching (PSM) to balance the covariates between the two groups. The propensity scores were created using a multivariate logistic regression model including age, sex, BMI, waist circumference (WC), eGFR, MI at index admission, numbers of coronary stents used, numbers of vessels intervened, income ≥ median, prior bleeding events, prior MI, prior LEAD, prior cerebrovascular events, prior hemiplegia, diabetes, hypertension, chronic kidney diseases, peptic ulcer, ESRD, and the CCI as covariates. The matching procedure was performed using the nearest neighbor method at a ratio of 1:5. The quality of the PSM was assessed using absolute standardized mean differences (SMDs).

Categorical variables were compared using a chi-square test, whereas continuous variables were compared using Student’s t-test or the Mann–Whitney U test. A Kaplan–Meier survival analysis with a log-rank test was utilized to compare the cumulative incidences of the clinical outcomes, including all-cause death, CV death, MACEs, ESRD, amputation and PTA after discharge between the PTA + PCI group and the PCI-only group. Cox proportional hazard models were used to assess the association of concomitant PTA with the clinical outcomes after index PCI. Multivariate Cox proportional hazard models were used to adjust the influences of confounding factors and reinforce the associations between concomitant PTAs and clinical outcomes. The multivariate models included age, sex, BMI, WC, MI at index admission, number of coronary stents used, income ≥ median, prior bleeding events, prior MI, prior LEAD, prior stroke, diabetes, hypertension, peptic ulcer, DAPT duration, and statin use as covariates. The full multivariate models were reduced through a backward variable selection process with a cutoff point of *p* > 0.05 to minimize the overfitting biases and multicollinearity among variables.

To estimate the influence of unmeasured confounders, *E*-values were estimated for the hazard ratio (HR) and 95% confidence interval (CI). The *E*-value is a simple and powerful sensitivity analysis tool to evaluate the strength of a risk-outcome association^10^. The *E*-value for an HR indicates the minimum HR that an unmeasured confounder should have with both the causal factor and outcome to completely explain away the apparent risk-outcome association, while the *E*-value for an upper or lower limit of CI indicates the minimum HR that the confounder should have with both the causal factor and outcome to make the CI include the null value. A high *E*-value indicates a strong causal relationship that would survive in the presence of a confounder having an HR < the *E*-value with both the causal factor and outcome.

Subgroup analyses were performed to detect the presence of differential impacts of concomitant PTA on the risk of MACEs, ESRD and limb events (the composite of amputation and PTA after discharge) in various subsets of patients. Mediation analyses were performed in the PSM cohort to identify the causal mediation effects of limb and renal outcomes on the association between concomitant PTA and all-cause death using a bootstrap resampling technique^11^. Parametric survival regression models with a Gaussian distribution, including the limb and renal outcomes as time-varying covariates, were used as the model objects for the mediators and the outcome in the mediation analyses. A mediation analysis for each mediator was performed in a set of 1000 bootstrap samples, and average total, direct and indirect causal mediation effects on the coefficients of the parametric survival regression models were reported.

All statistical analyses other than mediation analyses were conducted using the commercialized statistical software SAS 7.1 (SAS Institute, Cary, NC, USA). The mediation analyses were performed using statistical software R-4.3 (R Core Team, R Foundation for Statistical Computing, Vienna, Austria) and its packages “survival” and “mediation” in RStudio-1.3 (RStudio Team, RStudio Inc., Boston, MA, USA). A *p value* of < 0.05 was considered significant.

## Results

**Figure 1:**
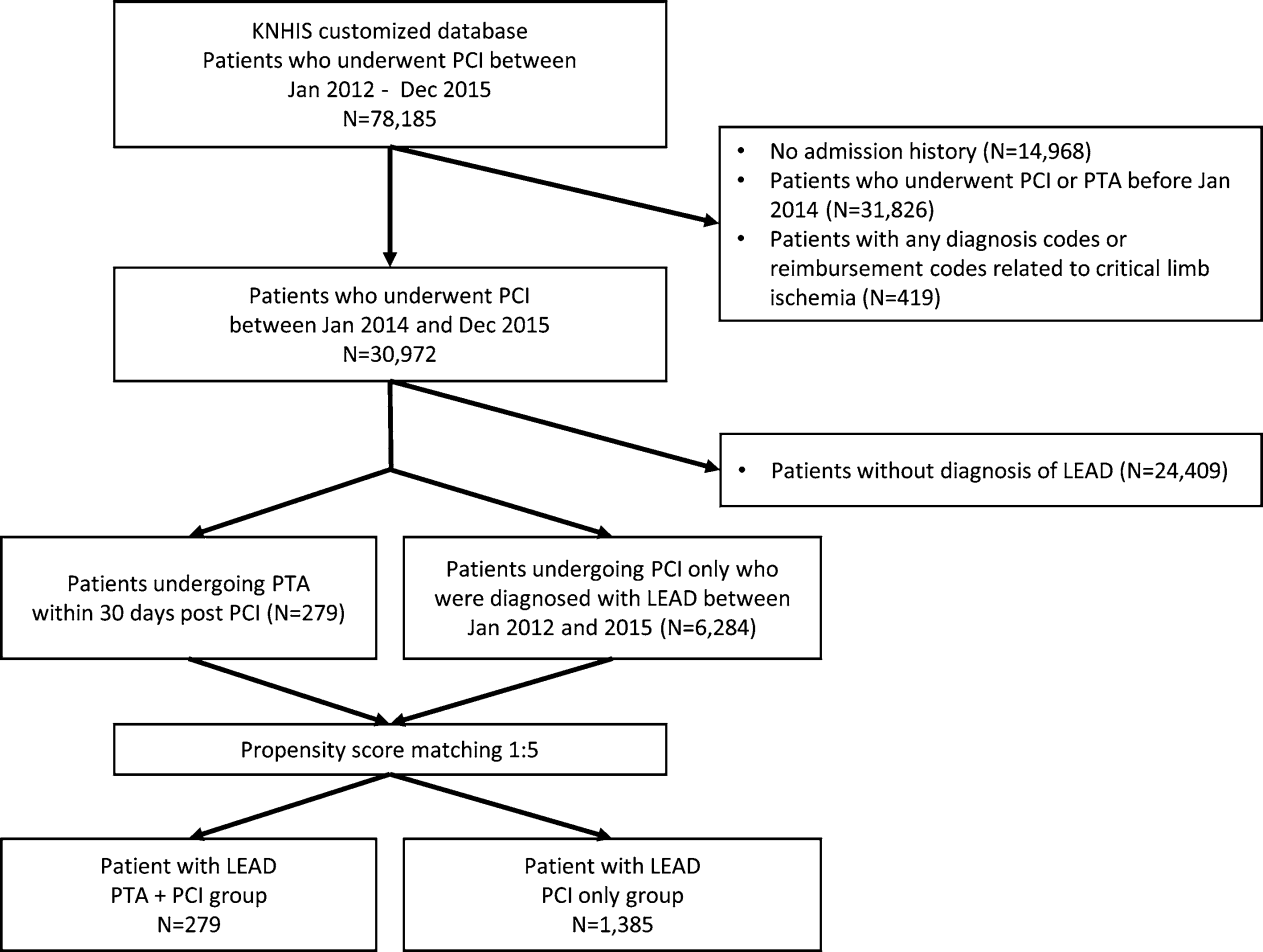
Patient selection process. Among the 30,972 patients who underwent PCI between Jan 2014 and Dec 2015, 6563 patients (279 for the PTA + PCI group; 6284 for the PCI only group) diagnosed with stable LEAD were analyzed. The baseline characteristics between the PTA + PCI group and the PCI only group were matched in an approximately 1:5 ratio through a PSM procedure.

Among 78,185 patients who underwent PCI and were registered in the KNHIS customized database, 6563 patients diagnosed with LEAD without any previous histories of foot ulcer or any limb ischemia-related diagnoses were included in the analyses (Fig. 1). Concomitant PTAs at the time of PCI were performed for 279 patients (PTA + PCI group), and PCI alone was performed for 6284 patients (PCI only group). After PSM was performed at a ratio of approximately 1:5, 1385 patients remained in the PCI-only group. The quality of the PSM procedure was described using absolute SMDs before and after matching in Supplementary Fig. 1.

The median follow-up duration was 3.78 (interquartile range [IQR] 3.24–4.34) years in the entire cohort (23,805.4 person-years) and 3.67 (IQR 3.16–4.22) years in the PSM cohort (5774.5 person-years). The follow-up duration was longer in the PCI-only group in the entire cohort (3.61 [IQR 3.09–4.31] vs. 3.79 [IQR 3.25–4.34]; *p* = 0.002) but was not significantly different between the groups in the PSM cohort (3.61 [IQR 3.09–4.31] vs. 3.68 [3.16–4.2]; *p* = 0.345).

The baseline characteristics before and after PSM are described in Table 1. In the unmatched cohort, the mean age and CCI score were higher and BMI, WC, eGFR, and DAPT duration were lower in the PTA + PCI group than in the PCI-only group. Male sex, multivessel CAD, the use of oral anticoagulants and comorbidities, including chronic lung disease, diabetes, ischemic stroke or cerebral ischemia, hemiplegia, CKD and ESRD, were more frequent, whereas MI at index PCI and previous histories of MI and stable LEAD were less frequent in the PTA + PCI group than in the PCI-only group. In the matched cohort, all baseline characteristics were evenly balanced between the two groups with SMD < 0.1, except for DAPT duration and the frequency of statin use, which were marginally lower in the PTA + PCI group.

**Table 1:** Baseline characteristics of study population before and after propensity score matching.

	Before PSM			After PSM			
	PTA + PCI	PCI only	p value	PTA + PCI	PCI only	p value	SMD
	N = 279	N = 6284		N = 279	N = 1385		
Male	214 (76.7)	3749 (59.7)	< 0.001	214 (76.7)	1089 (78.6)	0.527	− 0.042
Age (years)	70.8 ± 9.0	69.4 ± 10.0	0.011	70.8 ± 9.0	70.8 ± 9.4	0.998	< 0.001
Income levels ≥ median	134 (48)	3146 (50.1)	0.546	134 (48)	663 (47.9)	0.999	− 0.003
BMI (kg/m^2^)	23.8 ± 2.4	24.6 ± 2.6	< 0.001	23.8 ± 2.4	23.8 ± 2.4	0.989	0.001
WC (cm)	84.9 ± 6.5	85.6 ± 7.1	0.091	84.9 ± 6.5	85.1 ± 6.9	0.705	− 0.025
eGFR (mL/min/1.73m^2^)	70.4 ± 20.2	75.2 ± 23.7	< 0.001	70.4 ± 20.2	70.3 ± 21.3	0.98	0.002
Index MI	51 (18.3)	1958 (31.2)	< 0.001	51 (18.3)	250 (18.1)	0.996	− 0.005
Multi vessel CAD	53 (19)	1025 (16.3)	0.271	53 (19)	254 (18.3)	0.862	− 0.017
**Procedural characteristics**							
Number of coronary stents	1.97 ± 0.31	1.97 ± 0.25	0.795	1.97 ± 0.31	1.97 ± 0.25	0.992	0.022
Number of PCI vessels	1.24 ± 0.53	1.18 ± 0.46	0.099	1.24 ± 0.53	1.21 ± 0.50	0.464	0.049
Number of peripheral stents	1.71 ± 0.48	0.00 ± 0.00	–	1.71 ± 0.48	0.00 ± 0.00	–	–
**Types of PTA**						–	–
Plain balloon angiography	58 (20.8)	0 (0.0)		58 (20.8)	0 (0.0)		
Drug-eluting balloon	5 (1.8)	0 (0.0)		5 (1.8)	0 (0.0)		
Stenting	189 (67.7)	0 (0.0)		189 (67.7)	0 (0.0)		
Others	27 (9.7)	6284 (100.0)		27 (9.7)	1385 (100.0)		
**Types of PCI**			0.233			0.435	− 0.071
Plain balloon angiography	16 (5.7)	258 (4.1)		16 (5.7)	60 (4.3)		
Drug-eluting balloon	8 (2.9)	106 (1.7)		8 (2.9)	24 (1.7)		
Stenting	250 (89.6)	5826 (92.7)		250 (89.6)	1277 (92.2)		
Others	5 (1.8)	94 (1.5)		5 (1.8)	24 (1.7)		
DAPT duration (months)*	7 [1, 20]	12 [3, 28]	< 0.001	7 [1, 20]	10 [2, 26]	0.019	− 0.109
**Medication**							
DAPT	233 (83.5)	5515 (87.8)	0.044	233 (83.5)	1195 (86.3)	0.265	− 0.079
Statin	238 (85.3)	5708 (90.8)	0.003	238 (85.3)	1240 (89.5)	0.052	− 0.131
Oral and coagulants	14 (5.0)	169 (2.7)	0.034	14 (5)	52 (3.8)	0.413	0.066
**Charlson's comorbidity index**	5.3 ± 2.4	4.8 ± 2.5	< 0.001	5.3 ± 2.4	5.3 ± 2.7	0.889	0.009
≤ 3	75 (26.9)	2133 (33.9)	0.003	75 (26.9)	383 (27.6)	0.948	0.021
4–6	118 (42.3)	2716 (43.2)		118 (42.3)	587 (42.4)		
≥ 7	86 (30.8)	1435 (22.8)		86 (30.8)	415 (30.0)		
**Comorbidities**							
Any history of bleeding	31 (11.1)	588 (9.4)	0.381	31 (11.1)	130 (9.4)	0.437	− 0.057
Peptic ulcer	48 (17.2)	1047 (16.7)	0.876	48 (17.2)	236 (17)	1.000	− 0.004
Chronic lung diseases	42 (15.1)	662 (10.5)	0.022	42 (15.1)	205 (14.8)	0.987	0.008
Connective tissue disease	4 (1.4)	59 (0.9)	0.606	4 (1.4)	12 (0.9)	0.583	0.052
Heart failure	101 (36.2)	1948 (31.0)	0.075	101 (36.2)	489 (35.3)	0.784	0.019
Hypertension	256 (91.8)	5746 (91.4)	0.939	256 (91.8)	1269 (91.6)	1.000	− 0.005
Diabetes	219 (78.5)	4549 (72.4)	0.030	219 (78.5)	1106 (79.9)	0.665	0.032
Myocardial infarction	79 (28.3)	2434 (38.7)	0.001	79 (28.3)	390 (28.2)	1.000	− 0.003
Peripheral artery diseases	146 (52.3)	4146 (66)	< 0.001	146 (52.3)	733 (52.9)	0.908	0.012
Intracranial hemorrhage	4 (1.4)	63 (1.0)	0.533	4 (1.4)	17 (1.2)	0.768	0.019
Stroke or cerebral ischemia	107 (38.4)	1590 (25.3)	< 0.0001	107 (38.4)	513 (37.0)	0.684	0.028
Hemiplegia	17 (6.1)	109 (1.7)	< 0.001	17 (6.1)	59 (4.3)	0.238	− 0.095
Dementia	13 (4.7)	294 (4.7)	1.000	13 (4.7)	94 (6.8)	0.235	− 0.101
Chronic kidney diseases	40 (14.3)	491 (7.8)	< 0.001	40 (14.3)	188 (13.6)	0.808	− 0.024
End-stage renal diseases	18 (6.5)	178 (2.8)	0.001	18 (6.5)	82 (5.9)	0.840	− 0.025

Data are presented with the mean ± SD or N (%).

PSM, propensity-score matching; PCI, percutaneous coronary intervention; PTA, Percutaneous transluminal angioplasty; BMI, body mass index; WC, waist circumference; eGFR, estimated glomerular filtration rate; MI, myocardial infarction; DAPT, dual-antiplatelet therapy.

*Variables with a skewed distribution are presented with the median [1st quartile value, 3rd quartile value].

**Figure 2:**
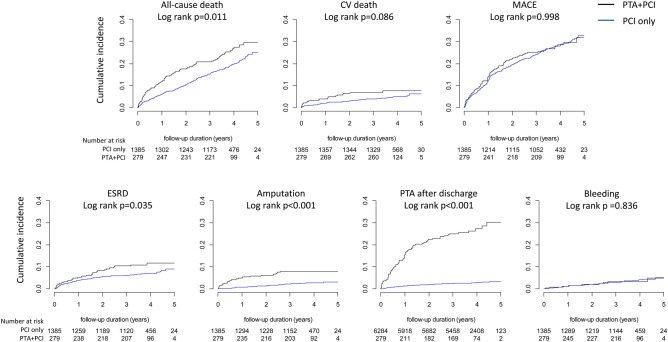
Cumulative incidences of clinical outcomes according to the treatment strategies. The cumulative incidences of all-cause death, ESRD, amputation and PTA after discharge were significantly higher in the PTA + PCI group than in the PCI only group, whereas there were no significant differences in the cumulative incidences of CV death and MACEs.

In the unmatched cohort, all clinical outcomes except MACEs occurred more frequently in the PTA + PCI group than in the PCI-only group (Supplementary Fig. 2). In the matched cohort, all-cause deaths, ESRD, amputation and PTA after discharge were more frequent in the PTA + PCI group than in the PCI-only group, whereas CV deaths were only marginally more frequent in the PTA + PCI group, and the cumulative incidence of MACEs was not different between the groups (Fig. 2).

**Figure 3:**
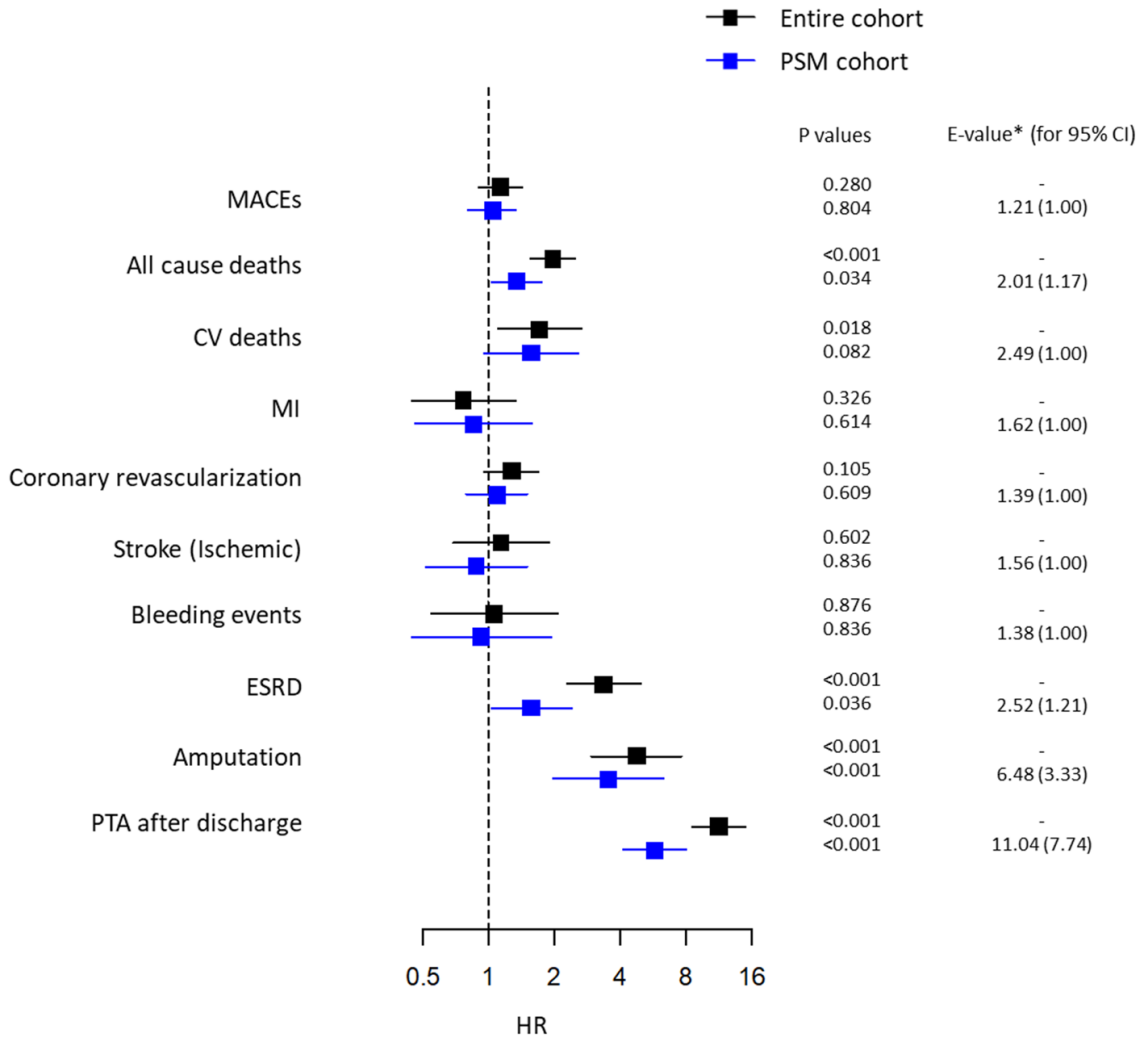
Univariate Cox proportional hazard models of the association of concomitant PTA at the time of PCI with clinical outcomes. Concomitant PTA was associated with higher risks of all-cause death, ESRD, amputation and PTA after discharge but was not associated with the risks of MACE, coronary revascularization, stroke or bleeding events in either the entire cohort or the PSM cohort. The concomitant PTA was significantly associated with a higher risk of CV deaths in the entire cohort but was not associated with the risk of CV deaths in the PSM cohort. *E-values indicate the HRs of unmeasured variables that could explain away the association between concomitant PTA and clinical outcomes and were estimated using the HRs and their lower limits of 95% CIs from the PSM cohort.

Univariate Cox proportional hazard models showed that the risks of all-cause death, ESRD, amputation and PTA after discharge were higher in the PTA + PCI group than in the PCI-only group, whereas the risks of MI, repeat coronary revascularization, ischemic stroke and bleeding events were not different between the two groups in either the unmatched or matched cohort (Fig. 3). The risk of CV death was higher in the PTA + PCI group than in the PCI-only group in the unmatched cohort, but the significance of the risk difference was only marginal in the matched cohort. The *E*-values for the HR and the lower limit of the 95% CI (LCI) were high for amputation and PTA after discharge and modest for all-cause death and ESRD but not significant for MACE, CV death, MI, repeat coronary revascularization, ischemic stroke or bleeding events in the matched cohort (Fig. 3 and Supplementary Table 4). In both the unmatched and matched cohorts, multivariate Cox proportional hazard models also showed associations of concomitant PTA with clinical outcomes, similar to those in the univariate models in the matched cohort (Table 2). As a sensitivity analysis, we excluded WC as a covariate from the PSM model and all multivariate Cox proportional hazard models; however, few changes were found in all associations except that the adjusted HR of concomitant PTA for CV death was significantly decreased in the PSM cohort (Supplementary Table 5).

**Table 2:** Multivariate Cox proportional hazard models for the association between concomitant PTA at the time of PCI and clinical outcomes.

Clinical outcomes	Before PSM				After PSM			
	Events		Models*		Events		Models*	
	N = 279	N = 6284	HR (95% CI)	P value	N = 279	N = 1385	HR (95% CI)	P value
Death	73 (26.2)	919 (14.6)	1.69 (1.32–2.15)	< 0.001	73 (26.2)	273 (19.7)	1.52 (1.17–1.98)	0.002
CV death	21 (7.5)	283 (4.5)	1.54 (0.99–2.42)	0.058	21 (7.5)	69 (5.0)	1.59 (0.97–2.59)	0.065
MACEs	79 (28.3)	1611 (25.6)	1.11 (0.89–1.40)	0.358	79 (28.3)	393 (28.4)	1.00 (0.78–1.27)	0.969
Myocardial infarction	13 (4.7)	414 (6.6)	0.87 (0.50–1.52)	0.625	13 (4.7)	80 (5.8)	0.85 (0.47–1.53)	0.591
Revascularization	49 (17.6)	962 (15.3)	1.25 (0.93–1.66)	0.136	49 (17.6)	237 (17.1)	1.09 (0.80–1.48)	0.594
Stroke	16 (5.7)	344 (5.5)	1.09 (0.66–1.81)	0.727	16 (5.7)	101 (7.3)	0.88 (0.52–1.50)	0.639
Any bleeding	9 (3.2)	209 (3.3)	0.97 (0.50–1.89)	0.920	9 (3.2)	49 (3.5)	0.91 (0.44–1.85)	0.787
ESRD	28 (10.0)	206 (3.3)	2.05 (1.37–3.06)	< 0.001	28 (10.0)	93 (6.7)	1.60 (1.05–2.45)	0.029
Amputation	20 (7.2)	108 (1.7)	3.81 (2.36–6.16)	< 0.001	20 (7.2)	34 (2.5)	3.41(1.96–5.92)	< 0.001
PTA after discharge	68 (24.4)	168 (2.7)	9.27 (6.93–12.4)	< 0.001	68 (24.4)	70 (5.1)	5.94 (4.25–8.30)	< 0.001

PSM, propensity score matching; PCI, percutaneous coronary intervention; PTA, Percutaneous transluminal angioplasty; CV cardiovascular; MACE, major adverse cardiovascular events; ESRD, endstage renal disease; HR, hazard ratio; CI, confidence interval.

*The multivariate model includes age, sex, BMI, waist circumference, MI at index admission, number of coronary stents used, income ≥ median, prior bleeding events, MI, PAD, stroke, diabetes, hypertension, peptic ulcer, DAPT duration and statin use as covariates. The model was reduced using a backward selection method (cutoff criterion, p > 0.1).

**Figure 4:**
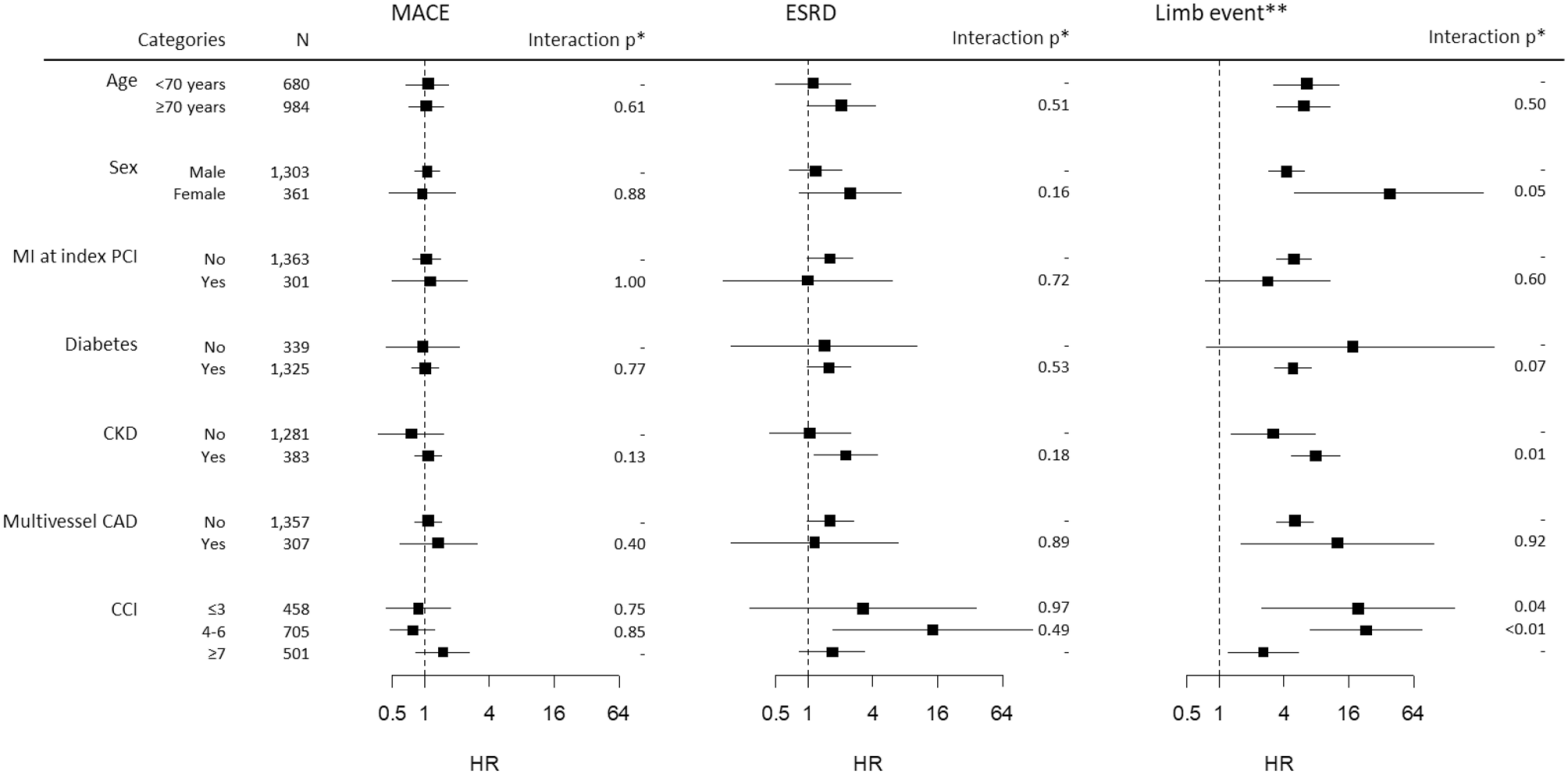
Subgroup analysis for the relationship between concomitant PTA at the time of PCI and clinical outcomes. The HRs and CIs were derived from univariate Cox proportional hazard models constructed in the PSM cohort. *p values for the interaction between the categories. For CCI, the *p value*s represent the interaction with the reference category (CCI ≤ 3). **Limb event is defined as a composite of amputation and PTA after discharge.

Subgroup analyses showed that the risks of MACE and ESRD were not different between the PTA + PCI group and the PCI-only group in all subgroups (Fig. 4 and Supplementary Table 6). However, the association of concomitant PTA with the risk of limb events was stronger in women, nondiabetic patients, patients with CKD and those with a low CCI (< 7).

**Figure 5:**
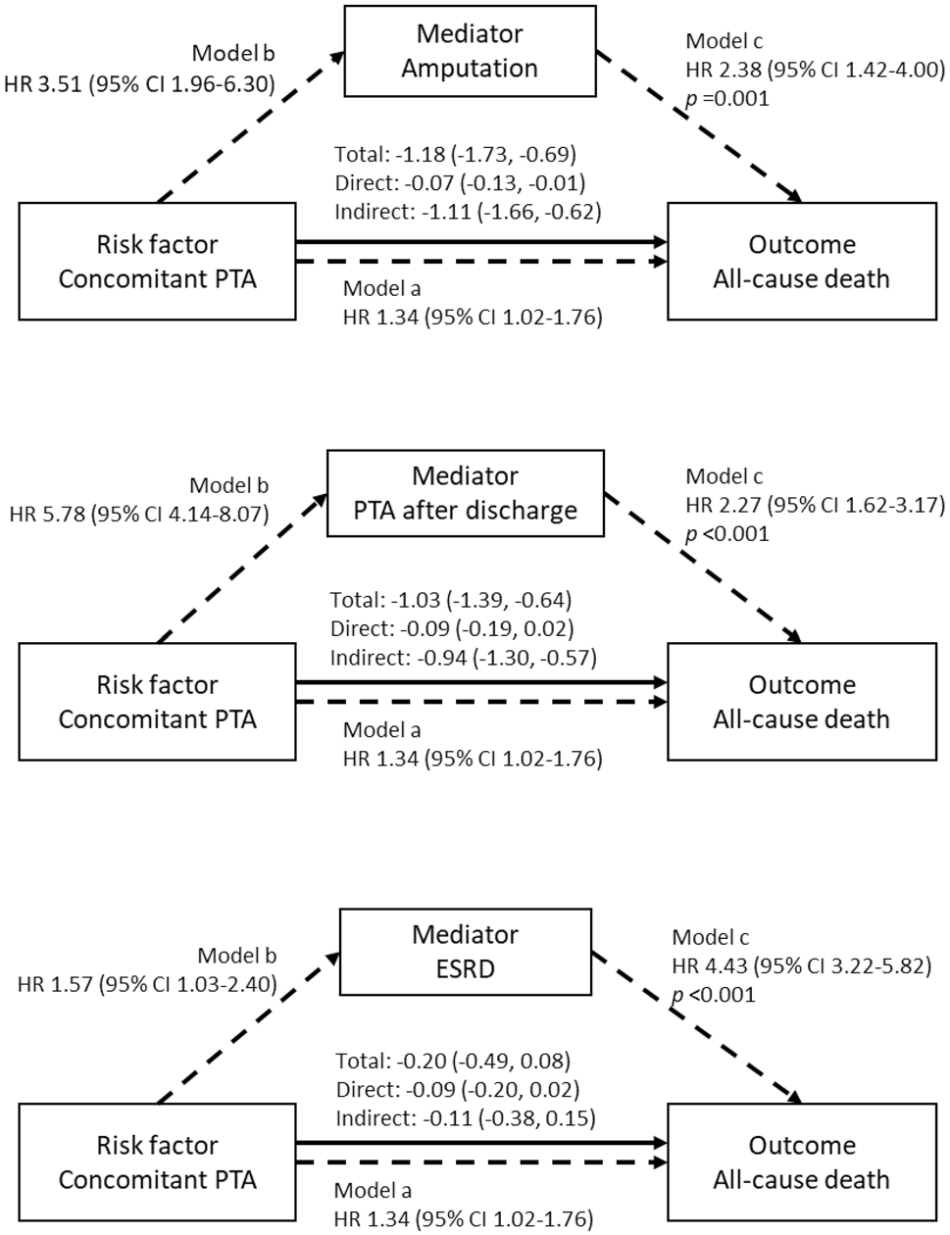
Mediation effects of amputation, PTA after discharge and ESRD on the association between concomitant PTA and all-cause death. Amputation and PTA after discharge significantly mediate the association between concomitant PTA and all-cause death, whereas ESRD does not mediate the association. The mediation analyses were performed in the PSM cohort using a bootstrap method. Statistical models used in the mediation analyses were time-varying survival regression models with a Gaussian distribution, while the associations presented in the diagrams were produced from Cox proportional hazard models (Model a and Model b) and time-varying Cox proportional hazard models (Model c).

Time-varying Cox proportional hazard models tested in the PSM cohort showed that amputation (HR 2.38; 95% CI 1.42–4.00), PTA after discharge (HR 2.27; 95% CI 1.62–3.17) and the development of ESRD (HR 4.43; 95% CI 3.22–5.82) during the follow-up period were associated with higher risks of all-cause death (Fig. 5). Mediation analyses revealed that undergoing amputation (93.9%; *p* < 0.001) and PTAs after discharge (91.7%; *p* < 0.001) strongly mediated the association between concomitant PTAs and the risk of all-cause death, whereas ESRD did not mediate the association (Fig. 5 and Supplementary Table 7).

## Discussion

In our results, we found that concomitant PTA at the time of PCI was associated with an increased risk of all-cause death, ESRD, amputation and PTA after discharge, whereas it was not associated with increased risks of CV deaths, repeat coronary revascularization, MI, stroke or bleeding events. Our results also showed that experiencing amputation and PTAs after discharge strongly mediated the association between concomitant PTAs and the risk of all-cause death.

Symptomatic LEAD commonly coincides with significant CAD; therefore, concomitant PTA is frequently performed in patients undergoing PCI as a default strategy worldwide. Bartus et al. reported a case series of 66 patients undergoing PTA and PCI during a single hospital stay and claimed that concomitant PTA was safe and should be encouraged to improve the patients’ quality of life^3^. A recent study by Koren et al. also reported in a small retrospective cohort that concurrent PTA had a similar 1-year MACE and mortality as staged PTA in patients undergoing PCI, advocating concurrent PTA^12^. However, concomitant PTA at the time of PCI has various concerns, including procedure complexity, lack of sufficient planning for the procedures, increase in contrast exposure and parenteral anticoagulant use, multiple punctures and higher restenosis rates of PTA. Therefore, the safety and efficacy of concomitant PTA should be more thoroughly investigated. Our results may provide some confidence in concomitant PTA regarding CV outcomes and bleeding but may imply potential hazards of concomitant PTA regarding all-cause deaths and unfavorable renal and limb outcomes, which demands further investigation. Our results are particularly concerning because the rates of revascularization and amputation have been reported as < 3%/year in patients with stable LEAD in a recent randomized controlled trial^13^ and in an observational study^14^, which are similar to those in the PCI-only group in our results (Fig. 2).

Similar rates of bleeding events and stroke between the groups are expected because parenteral anticoagulants would not have influenced the bleeding risk after discharge, and similar antiplatelet therapy regimens would have been used in both groups based on the recommendations from practice guidelines^1,15^. In contrast, the greater exposure to contrast media may have contributed to the higher incidences of ESRD in the PTA + PCI group. It has been established that the amount of contrast media exposure is associated with the risk of contrast-induced nephropathy^16^, and multiple studies have shown that contrast-induced nephropathy could cause permanent kidney damage and trigger progressive kidney dysfunction resulting in ESRD, especially in patients with prior CKD, who comprised approximately 14% of our study population^17,18^.

Because patients underwent PTA between 2014 and 2015 in our study, the majority of PTAs were performed using plain balloon angioplasty and BMS implantation, which exhibit ISR rates as high as 30–50% within a year^19,20^. These high ISR rates may have resulted in the higher incidences of PTA after discharge and amputation in the PTA + PCI group. The development of CLI has already been recognized as a predictor of death^21^. Repeat hospitalization and procedures for limb revascularization and wound care may be followed by a risk of procedural complications, malnutrition, infection, bleeding and thrombotic events, thus eventually leading to death. Our results also showed that experiencing PTAs after discharge or amputation significantly mediated the association between concomitant PTA and a higher risk of all-cause death. Although DES has been preferred to BMS in recent PTA, the ISR rate of DES still remains high for femoropopliteal lesions^22^. Therefore, the association between concomitant PTA and unfavorable limb outcomes may not have been different, although the study population included more patients undergoing PTA with DES.

The associations between concomitant PTA and unfavorable limb outcomes were stronger in the subgroups of women, CKD and CCIs < 7; this may also imply the role of ISR in worsening limb outcomes. Patients with CKD may have experienced limb events more frequently because they are predisposed to more severe LEAD and ISR^23^. Women may develop ISR more easily because of their smaller vessel diameter, although they have lower burdens of atherosclerosis than men^24^. However, in patients with fewer comorbidities, it is difficult to apply this reasoning to the results. Instead, frequent deaths may have interfered with the observation of limb events as a competing event in patients with a high CCI.

Since a high ISR rate would influence not only concomitant PTA but also other types of PTA, other factors may have been responsible for the unfavorable limb outcomes of concomitant PTA, unrelated to the high ISR rate. The use of larger contrast media volumes compared to the PCI-only group during index hospitalization, more frequent in-hospital bleeding events related to multiple endovascular procedures but not detected in our results and on-site PTA after lesion identification without sufficient preprocedural planning may have contributed to the unfavorable renal and limb outcomes of concomitant PTA. Although not confirmatory, our results suggest that concomitant PTA should be more selectively performed in patients with significant symptoms, only after sufficient procedure planning by an experienced vascular team, until more evidence supporting its safety and effectiveness are reported.

The concomitant PTA in our study included both PTA performed simultaneously with PCI as a single procedure and PTA performed as a staged procedure within the index hospitalization. We could not compare the clinical outcomes of simultaneous PTA with those of staged PTA; however, such comparisons would be an interesting subject for future randomized controlled trials because such trials will elucidate the unbiased impact of simultaneous PTA on clinical outcomes in terms of efficacy and safety and provide clear recommendations for performing PTA around the time of PCI.

The modest *E*-values for the LCI of the HRs for all-cause deaths and ESRD in the PSM cohort indicate that potential unmeasured confounders such as fragility might explain the association of concomitant PTA with these outcomes. In contrast, the *E*-values for the LCI of the HR for amputation and PTA after discharge were substantially high (3.33 and 7.75); thus, it is unlikely for any potential unmeasured factors to cancel the associations in the PSM cohort. Furthermore, to minimize the concern about the baseline difference in the severity of LEAD between the groups, we excluded patients with any conditions potentially related to acute limb ischemia, CLI and limb amputation and included patients with only stable LEAD who had not undergone any endovascular procedures during the preceding 3 years. In fact, a previous study showed that in patients with stable LEAD, an ankle-brachial index < 0.5 increased the risk of amputation and limb revascularization, but the adjusted HR of having an ankle-brachial index < 0.5 was only 1.96 and 2.69 for amputation and limb revascularization, respectively, which are smaller than the *E*-values observed in our results^25^.

### Limitations

This study has several limitations. First, since this study is observational, interpretation of the associations shown in the results as causality should be cautious. Although we adjusted confounders using multiple statistical methods and displayed the strength of the associations using the *E*-values, unmeasured confounders may still impact the associations shown in the results. In particular, symptom severity and lesion characteristics, including location, stenosis degree, calcification and tortuousness, were not investigated, but they may have significantly impacted the results as confounders. Moreover, the *E*-values for all-cause deaths and ESRD were small; thus, the associations may be explained by unmeasured confounders such as fragility or atherosclerosis burdens. Randomized controlled trials comparing clinical outcomes between concomitant PTA and delayed PTA in patients undergoing PCI are needed to confirm the causal associations. Second, since PTA was performed mainly with balloon angioplasty and BMS implantation in this study, it is unclear whether the results could be reproduced in populations where the use of DES prevailed. The unfavorable impact of concomitant PTA on limb events may be reduced in those populations. Third, even after PSM, the use of statins remained marginally more frequent in the PCI-only group (*p* = 0.052, SMD = − 0.13), which makes it difficult to exclude the influence of statins on the associations of concomitant PTA with clinical outcomes. Fourth, although this study was conducted using a nationwide insurance claim database, the number of patients included in the PTA + PCI group was only 279 because the enrollment period was only 2 years, and only 30% of the eligible patients were randomly selected for the study population because of the KNIHSS policy. Larger-scale investigations with longer enrollment periods are needed to fully reflect contemporary clinical practice. Fifth, our results did not include any data producing insights into the mechanisms behind the associations between concomitant PTA and the worse clinical outcomes. Finally, we did not investigate the quality of life, which is the most important reason for patients with stable LEAD to undergo concomitant PTA and it may have compensated for the unfavorable clinical outcomes.

## Conclusions

In patients with multisite artery diseases, including CAD and stable LEAD, concomitant PTA at the time of PCI is associated with increased risks of all-cause death, ESRD, repeat PTA and amputation. Experiencing PTA after discharge and amputation may lead to an increased risk of all-cause death in patients undergoing concomitant PTA. Our results suggest that concomitant PTA should be more cautiously selected as a treatment strategy for stable LEAD in patients undergoing PCI until more confirmatory evidence supports the procedure.

## Supplementary Information

Supplementary Information.

## Data Availability

The dataset was obtained from a data storage facility operated by the KNHISS (website: https://nhiss.nhis.or.kr/bd/ab/bdaba000eng.do), and the KNHIS and the Korean Ministry of Health and Welfare regulate access to this dataset. The data are accessible to any researchers under permission of the KNHISS granted after a review process for the researcher’s applications. A researcher will be provided with a dataset consisting of data from 30% of patients randomly selected from the entire Korean population eligible for the researcher’s study, according to the KNHISS research policy; therefore, a different researcher will receive a different dataset harboring equivalent results.

## Abbreviations

PTA: Percutaneous transluminal angioplasty
LEAD: Lower extremity artery disease
CV: Cardiovascular
CAD: Coronary artery disease
PCI: Percutaneous coronary intervention
ISR: In-stent restenosis
KNHIS: Korean National Health Insurance System
KNHISS: Korean National Health Insurance Data Sharing Service
WC: Waist circumference
CLI: Critical limb ischemia
eGFR: Estimated glomerular filtration rate
CKD: Chronic kidney disease
CCI: Charlson's comorbidity index
MI: Myocardial infarction
SMD: Standardized mean difference
MACEs: Major adverse cardiovascular events
ESRD: End-stage renal disease
BMS: Bare-metal stent
DES: Drug-eluting stent
PSM: Propensity score matching
LCI: Lower limit of the 95% confidence interval
