# Supplementary material for: Influence of concomitant percutaneous transluminal angioplasty with percutaneous coronary intervention on clinical outcomes of stable lower extremity artery diseases
Source: Sci Rep. 2022 Jul 29;12:12996. doi: 10.1038/s41598-022-16631-3 (PMC9338082; doi:10.1038/s41598-022-16631-3)
Supplement: Supplementary file 1 — Supplementary Information. [file 41598_2022_16631_MOESM1_ESM.docx]

**Supplementary Data**

**Supplementary data 1**

**Methods**

***Korean National Health Insurance Data Sharing Service (KNHISS) customized research database***

The KNHISS is a data sharing service operated by the Korean National Health Insurance System (KNHIS) to facilitate the use of National Health information for research purposes from various disciplines, providing a sample cohort database as well as customized databases. The KNHIS is a government-run single payer health care system covering 97% of the entire Korean population; other programs, including the Medical Assistance Program, cover the remaining 3% of the population. The KNHISS customized database includes insurance claim data from both patients covered by the KNHIS and those covered by the Medical Assistant Program and data from the National Health Screening.

Information regarding demographics, socioeconomic status, admission records, clinical diagnosis, comorbidities, medications and medical procedures was obtained from the insurance claim data. The earliest measurements of height, weight, waist circumference (WC) and serum creatinine levels following the index PCI were obtained from the National Health Screening database. Clinical outcomes were identified using the KCD-7 codes for the clinical diagnoses in the insurance claim database. The causes and dates of death were identified using KCD-7 codes and information on the discharge date obtained from the final admission during which death occurred.

**Supplementary Figures**


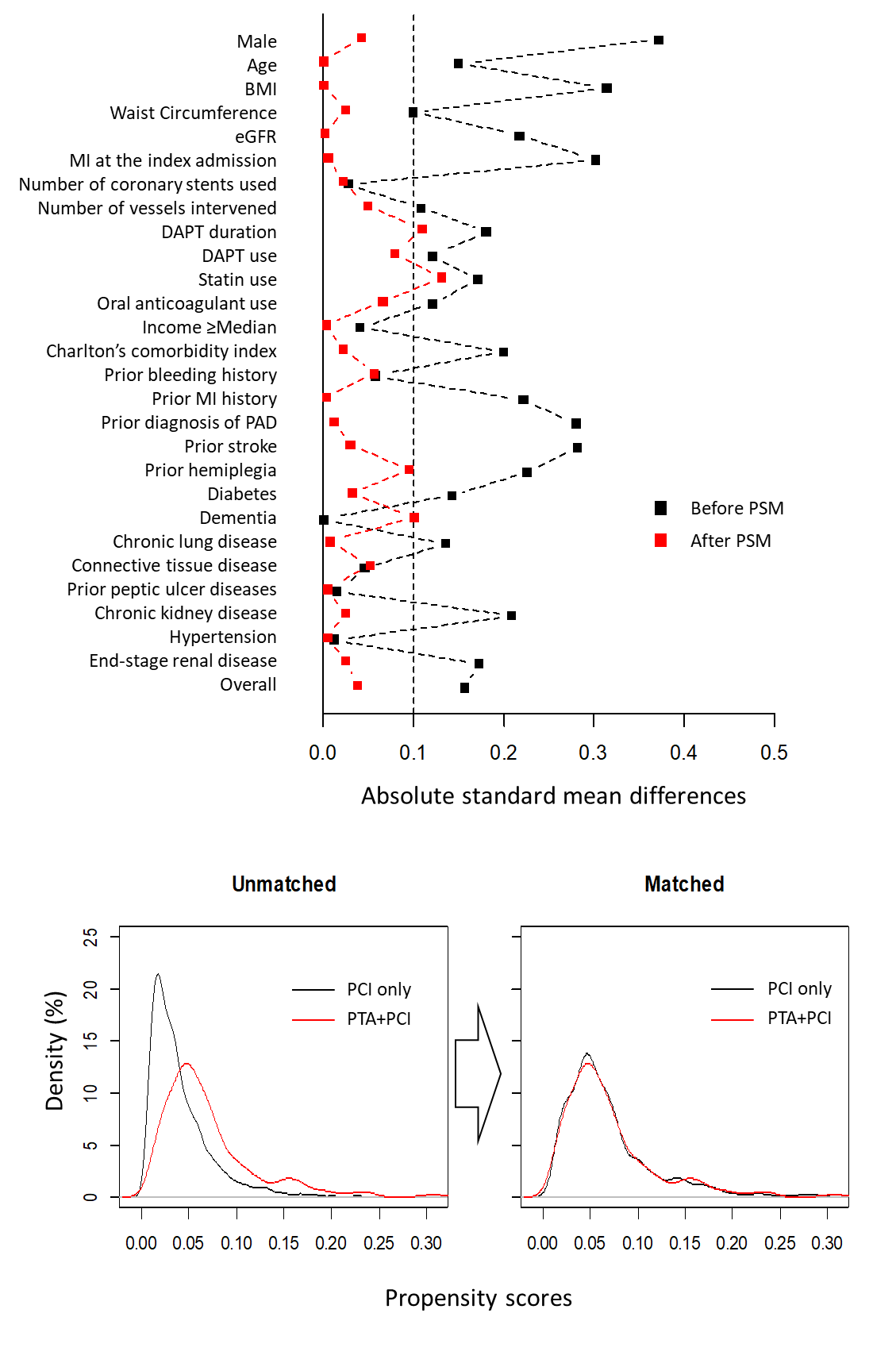


Supplementary figure 1. Quality of the PSM procedure.

Absolute standard mean differences were reduced to <0.1 in all variables (the upper panel) and the histograms of the propensity scores were overlapped (the lower panel) after PSM.


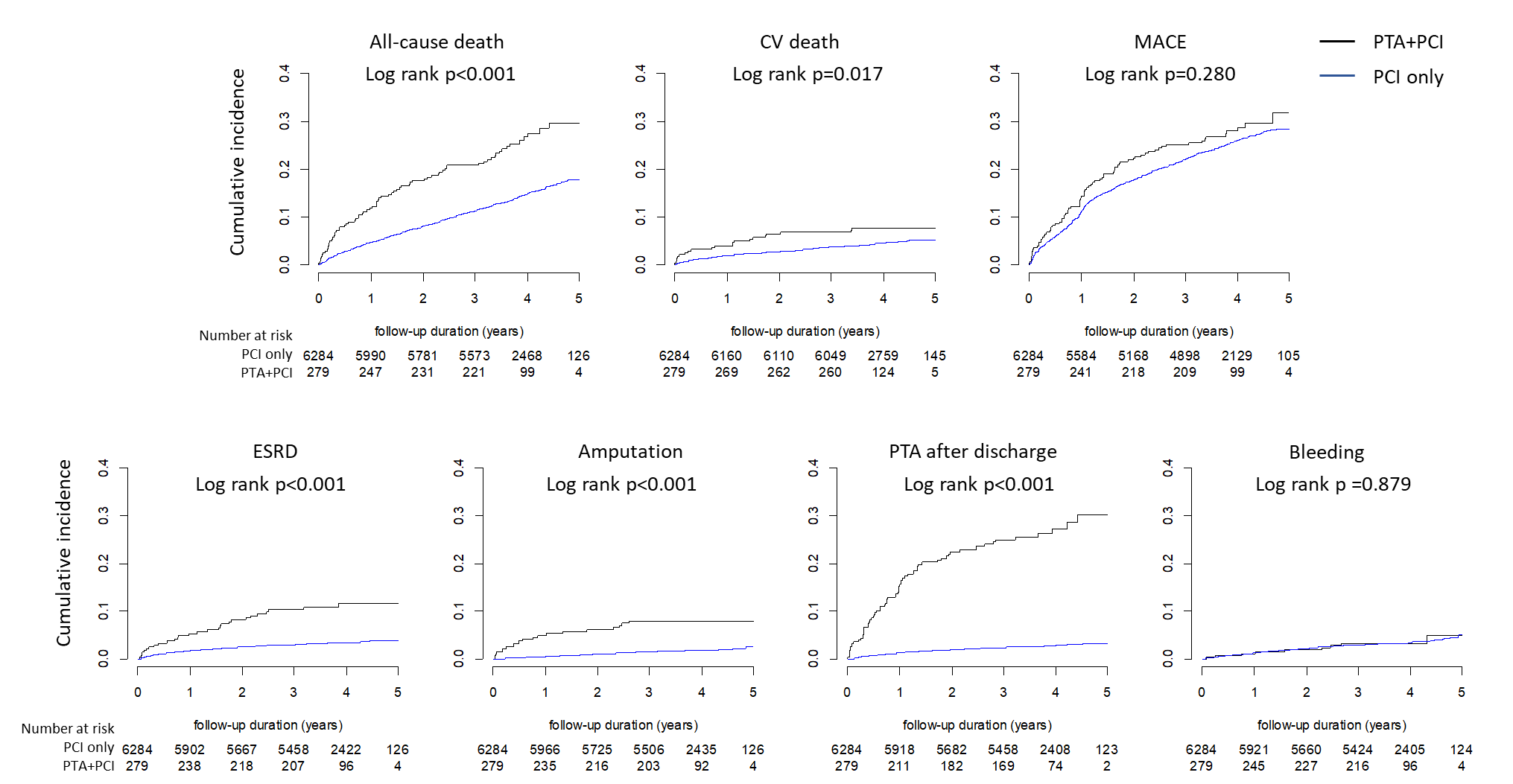


Supplementary figure 2. Cumulative incidences of clinical outcomes according to the treatments in the unmatched cohort.

All-cause death, CV death, the development of ESRD, amputation and PTA after discharge were more frequent in the PTA+PCI group than in the PCI only group, whereas the cumulative incidences of MACE and bleeding event were not different between the two groups.

**Supplementary Tables**

| Supplementary Table 1. The Korean Classification of Disease -7 Codes used to define the comorbidities and the Charlson's comorbidity index | | |
| --- | --- | --- |
|  | Clinical situations | Korean Classification of Disease -7 Codes |
| Comorbidities  or CCI parameters | Myocardial infarction | I21-I24 |
|  | Heart failure | I43, I50, I099, I110, I130, I132, I255, I420, I425, I426, I427, I428, I429, P290 |
|  | Peripheral artery diseases | I70-I71, K551, K558, K559, Z958, Z959, I731, I738, I739, I771, I790, I792 |
|  | Cerebrovascular diseases | G45, G46, I60, I61, I62, I63, I64, I65, I66, I67, I68, I69, H340 |
|  | Dementia | F00, F01, F02, F03, G30, G311, F051 |
|  | Hemiplegia | G81, G82, G041, G114, G801, G802, G830, G831, G832, G833, G834, G839 |
|  | Chronic lung diseases | J40, J41, J42, J43, J44, J45, J46, J47, J60, J61, J62, J63, J64, J65, J66, J67, I278, I279, J684, J701, J703 |
|  | Connective tissue diseases | M05, M06, M32, M33, M34, M315, M351, M353, M360 |
|  | Peptic ulcer diseases | K25, K26, K27, K28 |
|  | Mild liver diseases | B18, K73, K74, K700-K703, K709, K713-K715, K717, K760, K762 K763, K764, K768, K769, Z944 |
|  | Moderate to severe liver diseases | K704, K711, K721, K729, K765-K767 |
|  | Diabetes without complications | E100, E101, E106, E107, E109-E111, E116, E118-E121, E126, E129-E131, E136, E138-E141, E146, E148, E149 |
|  | Diabetes with complications | E102-E105, E112-E115, E107, E117, E122-E125, E127, E132-E135, E137, E142-E145, E147 |
|  | Chronic kidney diseases | N18, N19, I120, I131, N032-N037, N052-N057, N250, Z490-Z492, Z940, Z992 |
|  | End-stage renal disease | N18.5 |
|  | Hematologic malignancy | C00-C97 (except C77, C78, C79, C80) |
|  | Metastatic solid tumor | C77, C78, C79, C80 |
|  | Acquired Immunodeficiency syndrome | B20 B21, B22, B24 |
| Exclusion criteria/  outcomes | Diabetic foot ulcer | E1070, E1071, E1170, E1171, E1270, E1271, E1370, E1371, E1470, E1471, L97 |
|  | Amputation of extremities | E1070, E1071, E1170, E1171, E1270, E1271, E1370, E1371, E1470, E1471, L97 |
|  | Bleedings | I850, K221, K228, K252, K254, K256, K260, K262, K264, K266, K270, K272, K274, K276, K280, H052, K356, K431, J942, M250, K282, K284, K286, K290, K318, K552, K570-K575, K578, K579, K625, K661, K920, K921, K922, D62, R04 |
|  | Cardiovascular death | Death by I20-I74 |

| Supplementary Table 2. The National Health Insurance claim codes for PCI and CABG | | |
| --- | --- | --- |
|  | Claim codes | Procedure |
| PCI | M6561 | Elective PCI with coronary stent implantation (Single vessel) |
|  | M6562 | Elective PCI with coronary stent implantation (Multi-vessel) |
|  | M6563 | Elective PCI with coronary stent implantation+ atherectomy (Single vessel) |
|  | M6564 | Elective PCI with coronary stent implantation+ atherectomy (Multi-vessel) |
|  | M6565 | Primary PCI with coronary stent implantation for acute myocardial infarction |
|  | M6566 | Elective PCI with coronary stent implantation for chronic total occlusion |
|  | M6567 | Elective PCI with coronary stent implantation for chronic total occlusion + atherectomy |
|  | M6571 | Atherectomy for single vessel diseases |
|  | M6572 | Atherectomy for multi-vessel disease |
|  | M6551 | Elective PCI without coronary stent implantation (Single vessel) |
|  | M6552 | Elective PCI without coronary stent implantation (Multi-vessel) |
|  | M6553 | Primary PCI without coronary stent implantation for acute myocardial infarction |
|  | M6554 | Elective PCI without coronary stent implantation for chronic total occlusion |
| CABG | O1640 | CABG for 2 vessels |
|  | O1641 | CABG for 1 vessel |
|  | O1645 | CABG using autologous vessels |
|  | O1646 | CABG using artificial vessels |
|  | O1647 | Repeat CABG using autologous vessels |
|  | O1648 | CABG using autologous vessels for 3 vessels |
|  | O1649 | CABG using autologous vessels for 4 or more vessels |
|  | OA640 | Off-Pump CABG for 2 vessels |
|  | OA641 | Off-Pump CABG for 1 vessel |
|  | OA647 | Repeat Off-Pump CABG |
|  | OA648 | Repeat Off-Pump CABG for 3 vessels |
|  | OA649 | Repeat Off-Pump CABG for 4 or more vessels |
| PCI, percutaneous coronary intervention; CABG, coronary artery bypass graft | | |

| Supplementary Table 3. Insurance claim codes for the devices used in PCI and PTA | | |
| --- | --- | --- |
|  | Categories | Insurance claim codes |
| PCI | Coronary artery balloon | J4081, J8081 |
|  | Coronary drug-eluting stent* | J5083, J8083, J5084 |
|  | Coronary drug-coated balloon | J4080, J8080 |
|  | Others (bare-metal stents, cutting balloon, coronary stent graft) | J5231, J8231, J4082, J5603 |
| PTA | Peripheral artery balloon | J4071-J4073, J8071-J8074 |
|  | Peripheral artery drug-coated balloon | J4077 |
|  | Peripheral artery stent (bare-metal) | J5233-J5234, J8233 |
|  | Others (drug-coated stent, cutting balloon, stent graft beside aorta graft) | J5238, J5502-J5503, J8074, J4074 |
| * coronary drug-eluting stent includes bioabsorbable scaffold | | |
| PCI, percutaneous coronary intervention; PTA, percutaneous transluminal angioplasty | | |

| Supplementary Table 4. Associations of concomitant PTA at the time of PCI with clinical outcomes and the *E*-values. | | | | | | | | | | | |
| --- | --- | --- | --- | --- | --- | --- | --- | --- | --- | --- | --- |
|  |  |  | Univariate model | | | | Multivariate model* | | | | |
|  | Event (%) | | Statistics | | *E*-values | | Statistics | | *E*-values | | |
|  | PTA+PCI | PCI only | HR (95% CI) | P-value | for HR | for CI | HR (95% CI) | P-value | for HR | for CI |  |
| Before PSM | N=279 | N=6284 |  |  |  |  |  |  |  |  |  |
| Death | 73 (26.2) | 919 (14.6) | 1.96 (1.54-2.48) | <0.001 | 2.56 | 2.03 | 1.69 (1.32-2.15) | <0.001 | 2.77 | 1.97 |  |
| CV death | 21 (7.5) | 283 (4.5) | 1.71 (1.10-2.66) | 0.018 | 2.81 | 1.43 | 1.54 (0.99-2.42) | 0.058 | 2.45 | 1.00 |  |
| MACEs | 79 (28.3) | 1611 (25.6) | 1.13 (0.90-1.42) | 0.280 | 1.40 | 1.00 | 1.11 (0.89-1.40) | 0.358 | 1.46 | 1.00 |  |
| Myocardial infarction | 13 (4.7) | 414 (6.6) | 0.76 (0.44-1.32) | 0.326 | 1.96 | 1.00 | 0.87 (0.50-1.52) | 0.625 | 1.56 | 1.00 |  |
| Revascularization | 49 (17.6) | 962 (15.3) | 1.27 (0.95-1.69) | 0.105 | 1.64 | 1.00 | 1.25 (0.93-1.66) | 0.136 | 1.61 | 1.00 |  |
| Stroke | 16 (5.7) | 344 (5.5) | 1.14 (0.69-1.89) | 0.602 | 1.54 | 1.00 | 1.09 (0.66-1.81) | 0.727 | 1.41 | 1.00 |  |
| Any bleeding | 9 (3.2) | 209 (3.3) | 1.06 (0.54-2.05) | 0.876 | 1.31 | 1.00 | 0.97 (0.50-1.89) | 0.920 | 1.40 | 1.00 |  |
| ESRD | 28 (10.0) | 206 (3.3) | 3.37 (2.27-4.99) | <0.001 | 6.20 | 3.97 | 2.05 (1.37-3.06) | <0.001 | 3.51 | 2.08 |  |
| Amputation | 20 (7.2) | 108 (1.7) | 4.77 (2.96-7.68) | <0.001 | 9.01 | 5.37 | 3.81 (2.36-6.16) | <0.001 | 7.08 | 5.97 |  |
| PTA after discharge | 68 (24.4) | 168 (2.7) | 11.3 (8.52-15.0) | <0.001 | 22.10 | 16.52 | 9.27 (6.93-12.4) | <0.001 | 18.02 | 13.30 |  |
| After PSM | N=279 | N=1385 |  |  |  |  |  |  |  |  |  |
| Death | 73 (26.2) | 273 (19.7) | 1.34 (1.02-1.76) | 0.034 | 2.01 | 1.17 | 1.52 (1.17-1.98) | 0.002 | 2.41 | 1.62 |  |
| CV death | 21 (7.5) | 69 (5.0) | 1.56 (0.95-2.56) | 0.082 | 2.49 | 1.00 | 1.59 (0.97-2.59) | 0.065 | 2.56 | 1.00 |  |
| MACEs | 79 (28.3) | 393 (28.4) | 1.03 (0.81-1.32) | 0.804 | 1.21 | 1.00 | 1.00 (0.78-1.27) | 0.969 | 1.00 | 1.00 |  |
| Myocardial infarction | 13 (4.7) | 80 (5.8) | 0.85 (0.46-1.58) | 0.614 | 1.63 | 1.00 | 0.85 (0.47-1.53) | 0.591 | 1.63 | 1.00 |  |
| Revascularization | 49 (17.6) | 237 (17.1) | 1.09 (0.79-1.50) | 0.615 | 1.32 | 1.00 | 1.09 (0.80-1.48) | 0.594 | 1.32 | 1.00 |  |
| Stroke | 16 (5.7) | 101 (7.3) | 0.87 (0.51-1.49) | 0.608 | 1.56 | 1.00 | 0.88 (0.52-1.50) | 0.639 | 1.53 | 1.00 |  |
| Any bleeding | 9 (3.2) | 49 (3.5) | 0.93 (0.44-1.93) | 0.836 | 1.36 | 1.00 | 0.91 (0.44-1.85) | 0.787 | 1.43 | 1.00 |  |
| ESRD | 28 (10.0) | 93 (6.7) | 1.57 (1.03-2.40) | 0.036 | 1.52 | 1.21 | 1.60 (1.05-2.45) | 0.029 | 2.57 | 1.28 |  |
| Amputation | 20 (7.2) | 34 (2.5) | 3.51 (1.96-6.30) | <0.001 | 6.48 | 3.33 | 3.41(1.96-5.92) | <0.001 | 6.27 | 3.33 |  |
| PTA after discharge | 68 (24.4) | 70 (5.1) | 5.78 (4.14-8.07) | <0.001 | 11.04 | 7.75 | 5.94 (4.25-8.30) | <0.001 | 11.36 | 7.97 |  |
| PSM, propensity score matching; PCI, percutaneous coronary intervention; PTA, percutaneous transluminal angioplasty; CV cardiovascular; MACE, major adverse cardiovascular events; ESRD, end-stage renal disease; HR, hazard ratio; CI, confidence interval | | | | | | | | | | | |
| * The multivariate model includes age, sex, BMI, waist circumference, MI at index admission, number of coronary stents used, income ≥median, prior bleeding events, MI, PAD, stroke, diabetes, hypertension, peptic ulcer, DAPT duration and statin use as covariates. The model was reduced using a backward selection method (cut-off criterion, p>0.1). | | | | | | | | | | | |

| Supplementary Table 5. Multivariate Cox proportional hazard models without WC as a covariate for the association between the concomitant PTA and clinical outcomes. | | | | | | | | | |
| --- | --- | --- | --- | --- | --- | --- | --- | --- | --- |
|  | Before PSM | | | | After PSM | | | | |
|  | Events | | Models* | | Events | | Models* | | |
| Clinical outcomes | N=279 | N=6284 | HR (95% CI) | P value | N=279 | N=1385 | HR (95% CI) | P value |  |
| Death | 73 (26.2) | 919 (14.6) | 1.60 (1.26-2.04) | 0.0001 | 73 (26.2) | 273 (19.7) | 1.40 (1.08-1.82) | 0.0112 |  |
| CV death | 21 (7.5) | 283 (4.5) | 1.48 (0.94-2.31) | 0.0886 | 21 (7.5) | 69 (5.0) | 1.27 (0.78-2.06) | 0.3404 |  |
| MACEs | 79 (28.3) | 1611 (25.6) | 1.13 (0.90-1.41) | 0.3059 | 79 (28.3) | 393 (28.4) | 0.94 (0.74-1.19) | 0.5961 |  |
| Myocardial infarction | 13 (4.7) | 414 (6.6) | 0.91 (0.52-1.58) | 0.7262 | 13 (4.7) | 80 (5.8) | 0.77 (0.43-1.39) | 0.3901 |  |
| Revascularization | 49 (17.6) | 962 (15.3) | 1.27 (0.95-1.69) | 0.1099 | 49 (17.6) | 237 (17.1) | 1.06 (0.78-1.44) | 0.7062 |  |
| Stroke | 16 (5.7) | 344 (5.5) | 1.03 (0.62-1.70) | 0.9117 | 16 (5.7) | 101 (7.3) | 0.84 (0.50-1.43) | 0.5294 |  |
| Any bleeding | 9 (3.2) | 209 (3.3) | 1.00 (0.52-1.96) | 0.9901 | 9 (3.2) | 49 (3.5) | 0.80 (0.39-1.61) | 0.5239 |  |
| ESRD | 28 (10.0) | 206 (3.3) | 2.85 (1.92-4.24) | <.0001 | 28 (10.0) | 93 (6.7) | 1.64 (1.06-2.53) | 0.0249 |  |
| Amputation | 20 (7.2) | 108 (1.7) | 4.25 (2.63-6.86) | <.0001 | 20 (7.2) | 34 (2.5) | 2.80 (1.63-4.83) | 0.0002 |  |
| PTA after discharge | 68 (24.4) | 168 (2.7) | 10.0 (7.46-13.5) | <.0001 | 68 (24.4) | 70 (5.1) | 6.55 (4.64-9.25) | <.0001 |  |
| PSM, propensity score matching; PCI, percutaneous coronary intervention; PTA, Percutaneous transluminal angioplasty; CV cardiovascular; MACE, major adverse cardiovascular events; ESRD, end-stage renal disease; HR, hazard ratio; CI, confidence interval | | | | | | | | | |
| * The multivariate model includes age, sex, BMI, MI at index admission, number of coronary stents used, income ≥ median, prior bleeding events, MI, PAD, stroke, diabetes, hypertension, peptic ulcer, DAPT duration and statin use as covariates. The model was reduced using a backward selection method (cutoff criterion, p>0.1). | | | | | | | | | |

| Supplementary Table 6. Associations between concomitant PTA at the time of PCI and clinical outcomes in various subgroups | | | | | | | | | | | | |
| --- | --- | --- | --- | --- | --- | --- | --- | --- | --- | --- | --- | --- |
|  |  |  | MACE | | | ESRD | | | Amputation + PTA after discharge | | | |
| Group criteria | | N | event | HR (95% CI)* | p** | event | HR (95% CI)* | p** | event | HR (95% CI)* | p** |  |
| Age | <70 years | 680 | 213 (31.3) | 1.07 (0.67-1.71) | 0.61 | 64 (9.4) | 1.12 (0.50-2.51) | 0.51 | 75 (11.0) | 6.52 (3.25-13.1) | 0.50 |  |
|  | ≥70 years | 984 | 259 (26.3) | 1.05 (0.72-1.51) |  | 57 (5.8) | 2.03 (0.98-4.20) |  | 90 (9.1) | 6.15 (3.45-11.0) |  |  |
| Sex | Male | 1303 | 375 (28.8) | 1.06 (0.80-1.42) | 0.88 | 88 (6.8) | 1.17 (0.67-2.04) | 0.16 | 129 (9.9) | 4.24 (2.85-6.32) | 0.05 |  |
|  | Female | 361 | 97 (26.9) | 0.95 (0.46-1.98) |  | 34 (9.4) | 2.46 (0.83-7.34) |  | 36 (10.0) | 38.4 (5.06-290) |  |  |
| MI at index PCI | No | 1363 | 375 (27.5) | 1.04 (0.78-1.39) | 1.00 | 101 (7.4) | 1.61 (0.99-2.62) | 0.72 | 139 (10.2) | 4.97 (3.41-7.25) | 0.60 |  |
|  | Yes | 301 | 97 (32.2) | 1.13 (0.51-2.53) |  | 20 (6.6) | 1.00 (0.16-6.14) |  | 26 (8.6) | 2.85 (0.75-10.9) |  |  |
| Diabetes | No | 339 | 92 (27.1) | 0.96 (0.44-2.10) | 0.77 | 9 (2.7) | 1.41 (0.19-10.3) | 0.53 | 17 (5.0) | 17.4 (0.76-402) | 0.07 |  |
|  | Yes | 1325 | 380 (28.7) | 1.02 (0.76-1.36) |  | 112 (8.5) | 1.56 (0.97-2.51) |  | 66 (5.0) | 4.89 (3.34-7.16) |  |  |
| CKD | No | 1281 | 346 (27.0) | 0.75 (0.37-1.53) | 0.13 | 48 (3.7) | 1.04 (0.44-2.48) | 0.18 | 74 (5.8) | 3.18 (1.29-7.85) | 0.01 |  |
|  | Yes | 383 | 126 (32.9) | 1.07 (0.80-1.45) |  | 73 (19.1) | 2.24 (1.12-4.47) |  | 91 (23.8) | 7.90 (4.71-13.2) |  |  |
| multi-vessel CAD | No | 1357 | 376 (27.7) | 1.08 (0.80-1.44) | 0.40 | 99 (7.3) | 1.61 (0.97-2.67) | 0.89 | 128 (9.4) | 5.07 (3.38-7.59) | 0.92 |  |
|  | Yes | 307 | 96 (31.3) | 1.35 (0.59-3.13) |  | 22 (7.2) | 1.16 (0.19-7.03) |  | 37 (12.1) | 12.6 (1.60-99.3) |  |  |
| CCI | ≤3 | 458 | 115 (25.1) | 0.88 (0.44-1.73) | ref | 9 (2.0) | 3.24 (0.29-36.6) | ref | 18 (3.9) | 19.7 (2.48-156) | ref |  |
|  | 4-6 | 705 | 195 (27.7) | 0.78 (0.48-1.26) | 0.87 | 21 (3.0) | 14.4 (1.71-121) | 0.71 | 61 (8.7) | 23.3 (7.07-77.0) | 0.80 |  |
|  | ≥7 | 501 | 165 (32.9) | 1.48 (0.83-2.65) | 0.75 | 91 (18.2) | 1.68 (0.83-3.40) | 0.97 | 86 (17.2) | 2.58 (1.23-5.41) | 0.04 |  |
| * Univariate Cox proportional hazard models in each subgroup of propensity score-matched cohort.  ** P-values for the interaction between the categories in each subgroup. For CCI, p-values stand for the interaction of the categories with the reference category (CCI ≤3).  MACE, major adverse cardiovascular events; ESRD, end-stage renal disease; PTA, percutaneous transluminal angioplasty; HR, hazard ratio; MI, myocardial infarction; PCI, percutaneous coronary intervention; CKD, chronic kidney disease; CAD, coronary artery diseases; CCI, Charlson's comorbidity index | | | | | | | | | | | | |

| Supplementary Table 7. Model summary for mediators of the association between the concomitant PTA and all-cause death | | | | | | | |
| --- | --- | --- | --- | --- | --- | --- | --- |
|  |  |  |  |  | Mediation analysis summary | | |
| Mediators | Model outcome | Covariates | Estimates (95% CI) | p-value | Effects | Estimate (95% CI) | p-value |
| PTA after discharge | All-cause death | concomitant PTA | -0.083 (-0.187 - 0.021) | 0.120 | Total effect | -1.03 (-1.39 --0.64) | <0.001 |
|  |  | PTA after discharge | 1.519 ( 1.467 - 1.746) | <0.001 | Direct effect | -0.09 (-0.19 - 0.02) | 0.120 |
|  |  | Log(scale) | -0.495 (-0.420 --0.570 | <0.001 | Indirect effect | -0.94 (-1.30 --0.57) | <0.001 |
|  | PTA after discharge | concomitant PTA | -0.589 (-0.808 --0.370) | <0.001 | % mediated | 91.7% (81.6% - 100%) | <0.001 |
|  |  | Log(scale) | -0.108 (-0.191 --0.025) | 0.011 |  |  |  |
| Amputation | All-cause death | concomitant PTA | -0.070 (-0.129 --0.010) | 0.022 | Total effect | -1.18 (-1.73 --0.69) | <0.001 |
|  |  | Amputation | 1.869 ( 1.738 - 2.000) | <0.001 | Direct effect | -0.07 (-0.13 --0.01) | 0.012 |
|  |  | Log(scale) | -1.068 (-1.143 --0.992) | <0.001 | Indirect effect | -1.11 (-1.66 --0.62) | <0.001 |
|  | Amputation | concomitant PTA | -0.593 (-0.874 --0.312) | <0.001 | % mediated | 93.9% (87.0% - 99%) | <0.001 |
|  |  | Log(scale) | -0.253 (-0.372 --0.134) | <0.001 |  |  |  |
| ESRD | All-cause death | concomitant PTA | -0.091 (-0.201 - 0.018) | 0.100 | Total effect | -0.20 (-0.49 - 0.08) | 0.190 |
|  |  | ESRD | 1.083 ( 0.933 - 1.231) | <0.001 | Direct effect | -0.09 (-0.20 - 0.02) | 0.120 |
|  |  | Log(scale) | -0.457 (-0.533 --0.382) | <0.001 | Indirect effect | -0.11 (-0.38 - 0.15) | 0.420 |
|  | ESRD | concomitant PTA | -0.098 (-0.346 - 0.150) | 0.438 | % mediated | 59.7% (-231% - 371%) | 0.250 |
|  |  | Log(scale) | -0.150 (-0.237 --0.063) | <0.001 |  |  |  |
| All models are time-varying survival regression models (Gaussian) | | | | | | | |
